# The effects of gallic acid on pain and memory following transient global ischemia/reperfusion in Wistar rats

**Published:** 2013

**Authors:** Yaghoob Farbood, Alireza Sarkaki, Sheida Hashemi, Mohammad Taghi Mansouri, Mahin Dianat

**Affiliations:** 1 *Physiology Research Center and Department of Physiology, Faculty of Medicine, Ahvaz Jundishapur University of Medical Sciences, Ahvaz, I. R. Iran*; 2 *Physiology Research Center, Medicinal Plants Research Center and Department of Physiology, Faculty of Medicine, Ahvaz Jundishapur University of Medical Sciences, Ahvaz, I. R. Iran *; 3 *Physiology Research Center and Department of Pharmacology, Faculty of Medicine, Ahvaz Jundishapur University of Medical Sciences, Ahvaz, I. R. Iran*

**Keywords:** Cerebral Ischemia/Reperfusion, Gallic Acid, Memory, Pain, Rats

## Abstract

**Objective:** It is generally agreed that most of the phenomena observed during brain ischemia and reperfusion can be explained by the damage to membrane structure. Oxidative stress is resulted in an imbalance between high consumption of oxygen and low levels of endogenous antioxidants. It is known that gallic acid (GA) is a strong antioxidant. The present study was carried out to evaluate the effect of GA on ischemia/reperfusion (I/R)-induced brain injury in rats.

**Materials and** **Methods:** Wistar adult male rats weighing 200–250 g were divided into six groups as sham operated (Sh), ischemia/reperfusion (I/R) received normal saline (I+Veh), I/R groups treated with gallic acid (I+GA, 50, 100, or 200 mg/kg, orally, respectively), or with 100 mg /kg phenytoin (I+Phen). The global cerebral I/R injury was induced by occluding bilateral common carotid arteries (BCCA) for 20 min, followed by 5 days reperfusion in adult male rats.

**Results:** It was found that administration of 100 mg/kg GA for 5 days before and 5 days after I/R induction reversed gait performance, sensorimotor disorders (p<0.01), and hypoalgesia (p<0.001) while dose of 50 mg/kg increased passive avoidance memory significantly (p<0.05).

**Conclusion:** Our findings clearly demonstrate that GA has beneficial effects on behavioral impairments after brain injury induced by I/R. The results of this study show that GA pretreatment ameliorates cerebral ischemia/reperfusion injury and enhances the antioxidant defense against BCCA occlusion-induced I/R in rats, so it exhibits cerebroprotective property.

## Introduction

Different studies have shown that cerebral ischemia most commonly occurs in patients with stroke (Imai et al., 2007 ▶; Li et al., 2011 ▶). Disorders of the cerebral circulation are associated with neurological and psychiatric illnesses. Clinical evidence supports the hypothesis that chronic cerebral hypoperfusion is associated with pain disorder and cognitive decline, both in aging and in neurodegenerative disorders (Kalaria 2000 ▶; Sopala and Danysz 2001 ▶). Transient global cerebral ischemia causes loss of pyramidal cells in CA1 region of hippocampus and cerebral cortex, striatum, cerebral cortex, and thalamus (Freund et al., 1990 ▶; Imai et al., 2007 ▶; Sharifi et al., 2012 ▶). It also impairs cognition in humans (Jokinen et al., 2006 ▶) and behavioral performance associated with cognitive and motor disorders in rodents (Langdon et al., 2008 ▶). Ischemia-induced neuronal degeneration is also observed in other structures such as the striatum, cerebral cortex, and thalamus (Pulsinelli et al., 1982 ▶; Smith et al., 1984 ▶). 

Oxidative stress is believed to contribute to neuronal damage induced by cerebral ischemia/reperfusion (I/R) injury (Mansoorali et al., 2012 ▶). I/R leads to inflammation and oxidative stress which damages membrane highly polyunsaturated fatty acids (HPUFAs) and eventually induces neuronal death (Quartu et al., 2012 ▶). 

Free radical-induced oxidative damages of macromolecules and cell death are important factors in the pathogenesis of I/R brain injury (Dubinsky et al., 1995 ▶). The brain is particularly vulnerable to oxidative stress injury due to its high consumption of oxygen, abundant polyunsaturated fatty acids, and low levels of endogenous antioxidants (Margaill et al., 2005 ▶). Compelling evidence implicates free radicals as major contributors to ischemic tissue injury in the central nervous system (CNS). It has been well documented that ischemia increases lipid peroxidation and reactive oxygen species (ROS), which cause secondary neural tissue damage. Lipid peroxidation is an autocatalytic mechanism leading to oxidative destruction of cellular membranes (Cheeseman, 1993 ▶). 

The formation of these oxidation products in the human and animal brains increases with age and I/R (Chen et al., 1997). Therefore, it is interesting to study the effects of antioxidants, free radical scavengers, or trapping agents as potential cerebroprotective agents on various brain injuries including I/R-mediated brain injury.

GA, a polyphenyl class natural product from gallnut and green tea, is known to be antioxidant, anti-inflammatory, and radical scavenger. GA is an endogenous product found in plants (Kuhr and Engelhardt, 1991 ▶; Shahrzad and Bitsch, 1996 ▶; Singleton, 1981 ▶). It is widely distributed throughout the plant kingdom, where it is present either in free or bound forms found in tea leaves which is extracted in hot water infusions or more commonly as a constituent of tannins namely gallotannin (Niemetz and Gross, 2005 ▶). Strawberries, pineapples, bananas, lemons, red and white wines, gallnuts, sumac, witch hazel, tea leaves, oak bark, and apple peels are some of the natural products which are rich in gallic acid (Chu et al., 2002 ▶; Wolfe et al., 2003 ▶). Regarding its biological activity, gallic acid exerts anti-bacterial, anti-viral, anti-inflammatory, antioxidant, and antimelanogenic activities via inhibition of tyrosine’s activity (Kim et al., 2006 ▶; Wolfe et al., 2003 ▶).

 It also inhibits high fat diet-induced dyslipidaemia, anti-proliferative, pro-apoptotic, and anti-tumorigenic effects against prostate carcinoma xenograft growth in nude mice (Kaur et al., 2009 ▶). GA binds to proteins and key minerals such as iron, zinc, and calcium and affects their bioavailability by forming insoluble complexes (Niho, 2001 ▶). A significant increase of serum alanine aminotransferase, aspartate aminotransferase, and lactate dehydrogenase activities has been also observed. These are indicators of liver damage after acute ethanol consumption. GA therapy has significantly reduced the increase in these biomarkers, indicating a possible hepatoprotective effect of GA in a dose-dependent way. Gallic acid especially at 200 mg/kg improved ethanol-mediated pancreatic tissue damage. Gallic acid treatments decreased release of lysosomal cathepsin B and L enzymes into cytoplasmic fraction and prevented alcohol mediated pancreatic tissue injury. Preventive effect of gallic acid might be dose-dependent (Kanbak et al., 2012 ▶).

 GA is a strong antioxidant that possesses antimutagenic and anticarcinogenic activities (Inoue et al., 1994 ▶). Its derivative, 4-Omethylgallic acid (4OMGA), has been reported as the main metabolite of GA in rats (Zong et al., 1999 ▶) and humans (Shahrzad et al., 2001 ▶). Moreover, because hippocampal neuronal cell death is selectively vulnerable to global and focal ischemia, ischemic animal models have been widely used to investigate the mechanism of neuronal death and the action mechanisms of neuroprotective agents in vascular dementia (Gustavsson et al., 2005 ▶; Ylikoski and Hanninen 2003 ▶; Zarow et al., 2005 ▶).

Learning and memory deficits are also observed in global cerebral I/R (Zhao et al., 2007 ▶). By the way, ROS which causes vasospasms in the brain (Luo et al., 1995 ▶ ) is produced during reperfusion after ischemia and is very likely associated with pain. Therefore, in the present study it is interesting to study the effects of gallic acid on memory and pain after induction of global cerebral I/R in rats.

## Materials and Methods

Wistar adult male rats weighing 200-250 g were obtained from the main animal house of Ahvaz Jundishapur University of Medical Sciences (AJUMS). Animals were kept at constant room temperature under a 12 h light/dark cycle at least 15 days prior to begin the experiments. Commercial food pellets and tap water were freely available.

Animals were divided randomly into two main groups: A) Sh: sham operated and B) I/R: ischemic/reperfusion. I/R groups were again divided into 4 sub-groups with 16 rats in each including: 1) I+Veh: received normal saline (2 ml/kg, orally) and 2-4) I+GA groups received GA (50, 100, or 200 mg/kg, orally, respectively). The animals received GA or vehicle for 5 days before and also 5 days after I/R induction. 6) positive control group were I/R rats which received 100 mg/kg phenytoin intraperitoneally every 24 h for 48 h before and 48 h after induction of ischemia as a routine selecting drug used to care and treat cerebral ischemic patients in hospitals (Hosseinzadeh et al., 2005 ▶).

**Chemicals**

Gallic acid (3, 4, 5, trihydroxybenzoic acid, C6H2 (OH) 3-COOH, MW=170/12) was purchased from Sigma-Aldrich Co., USA. Phenytoin was obtained from Razi Pharmaceutical Co. Tehran, I.R. Iran. Phenytoin was suspended in 80 ml normal saline.

**Induction of global cerebral I/R**

We used Quartu’s method with little modification. Briefly, rats were anesthetized with ketamine/xylazine (50:5 mg/kg, i.p). A neck ventral midline incision was made and the common carotid arteries were then exposed and gently separated from the vagus nerve. Transient bilateral occlusion of common carotid artery (CCA) was performed with microvascular clamps and lasted 20 min (Kleinschnitz et al., 2013 ▶; Yigitkanli et al., 2013 ▶). Sham-operated animals (Sh) were under similar surgical procedures without carotid arteries occlusion (Leeuwenburgh and Heinecke, 2001 ▶). 

Behavioral assessment to prove the ischemic brain damage consisted sensorimotor (spontaneous activity, and symmetry in the movement of four limbs), gait, cognitive (passive avoidance task), and pain (tail flick reflex) evaluations. All the sham-operated (Sh) and I/R rats were assessed for sensorimotor and gait before surgery (baseline), and 5 days after surgery by an examiner who was blind to the type of surgical procedure. 

**Sensorimotor evaluation**

It consisted of two tests developed and described by Garcia (Garcia et al., 1995 ▶) with some modifications as described below. The scores assigned to each rat at the end of each examination is the sum of the two test scores. The minimum neurological score is three and the maximum is 4. I/R rats with at least score 3 in each test were selected in this study. 

**Spontaneous activity**

 The animal is observed for 5 min in its normal cage. Scores indicate the following: (1) rat moves around, explores the environment, and approaches at least three walls of the cage, (2) rat moves around in the cage but does not approach all sides and hesitates to move, although it eventually reaches at least one upper rim of the cage (height=10 cm), (3) rat dose not rise up at all and barely moves in the cage, and (4) rat does not move at all. 

**Symmetry in the movement of four limbs**

The rat is held in the air by the tail to observe symmetry in the movement of the four limbs. Scores indicate the following: (1) all four limbs extend symmetrically, (2) limbs on one side extend less or more slowly than those on the other side; or slow extension of the four limbs, (3) limbs on one or both sides show minimal movements, and (4) forelimbs on one or both sides do not move at all.

**Gait performance evaluation**

This was carried out by means of the elevated platform test (Wallace et al., 1980 ▶). Each rat was positioned at the beginning of a 5-cm wide, 60-cm long wood bridge suspended between two platforms. The rats were tested for their ability to remain on the bridge during a single 3-min trial. The number of rats falling from the bridge and the length of the bridge covered by each animal, either falling or not, was recorded. 

**Pain test**

A tail-flick assay was performed using the tail-flick analgesia meter and radiant heat tail flick. A beam of light was focused on the dorsal surface of the tail approximately 8 cm from the tail’s tip. The intensity of the heat stimulus was adjusted so that the baseline latencies settled on 10 s (Kaeidi et al., 2013 ▶; Li et al., 2013 ▶). The tail-flick latency was measured 5 days after induction of I/R in rats. Each nociceptive test consisted of the mean value of three measurements in each rat. 

**Passive avoidance task**

The apparatus used for evaluation of the passive avoidance task was two-way shuttle box (Borj Sanaat Co. Tehran, Iran), which consisted of two adjacent Plexiglas compartments of identical dimensions (27×14.5×14 cm) with grid floor. The floor of two compartments was covered with stainless steel bars (2 mm diameter) with 1 cm distance. Light compartment was illuminated by a 5 W lamp mounted on its wall just below a movable transparent Plexiglas ceiling. The Tamburella’s procedure with little modification was used for passive avoidance memory test (Tamburella et al., 2012 ▶). Each rat was allowed a 10 min adaptation period prior and free access to either the light or dark compartment of the box to avoid training and after being placed in a shuttle-box (in order to familiarize with the instruments). Following this adaptation period, on the second day (initial phase) rats were placed into the illuminated compartment and 30 sec later the sliding door was raised (initial latency was recorded as learning phase). Upon entering the dark compartment the door was closed and a 1.5 mA constant-current was applied to animal fore and hind paws for 3 seconds as electrical shock. After 20 sec (in order to consolidation) the rat was removed from the dark compartment and placed into the home cage.

 In order to test short-term, 24 h after receiving foot shock, the rats were placed in illuminated chamber again and 30 sec later the sliding door was raised and the latency of entering the dark compartment was recorded again as memory test (step-through latency). The maximum time that was considered in this procedure was 300 sec (Levy et al., 1985 ▶; Lipton and Rosenberg, 1994 ▶; Mahut et al., 1982 ▶).

**Statistical analysis**

Data were expressed as mean±SEM. of values for initial latency and memory tests. Statistical analyses were performed by paired t-test to compare initial and step-through latencies in each group, one-way ANOVA to compare the initial and step-through latencies for passive avoidance task in all groups that was followed by LSD post-hoc test, respectively. A p-value less than 0.05 was assumed to denote a significant difference.

## Results

**Motor evaluation**

Spontaneous activity and symmetry in the movement of four limbs were evaluated during 3 min in sham operated (Sh) and I/P received normal saline (I+Veh) groups. mean±SEM of spontaneous activity (3.1±0.1) and symmetry in the movement (3.5±0.05) scores in I+Veh group were increased significantly (p<0.01) compared with Sh group scores in similar examinations (1±0.1, 1±0.1). Numbers of falling and length of the bridge covered performances was evaluated in Sh and I+Veh groups. mean±SEM of numbers of falling in I+Veh group (4.75±0.3) was increased significantly (p<0.01) compared with Sh group (0.3±0.02). Length of the bridge covered in I+Veh group (0.5±0.01) was decreased significantly (p<0.01) compared with Sh group (3±0.01) in similar examinations. 

**Passive avoidance task**

**Figure 1:**
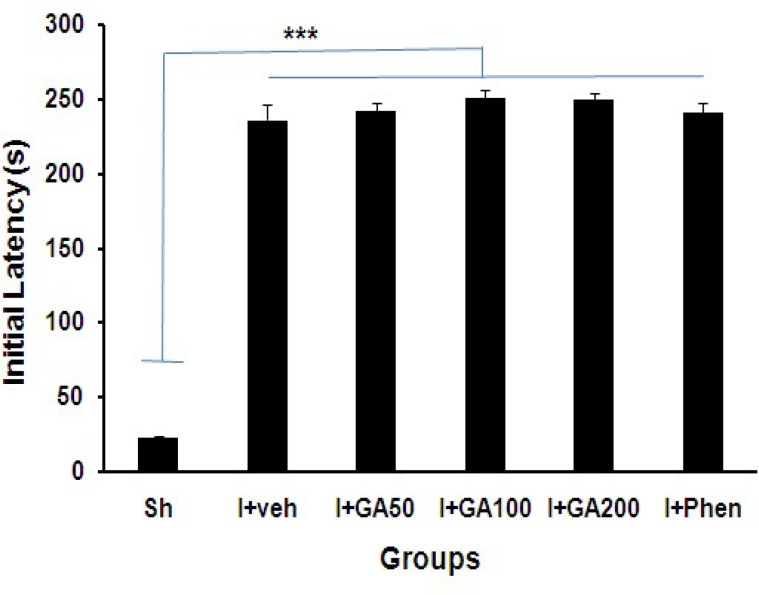
Initial latency (s) of sham operated (Sh) and I/R groups treated with normal saline, (I+Veh), different doses of gallic acid (I+GA50, I+GA100, and I+GA200) or phenytoin (I+Phen). Data are presented as mean± SEM. ***p<0.001 to compare with Sh group. One-way ANOVA followed by LSD post-hoc test, n=16 in each group

**Figure 2:**
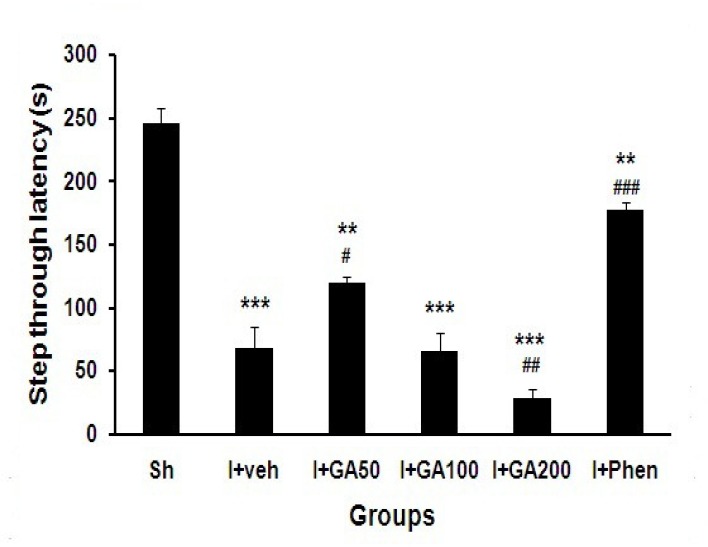
Step through latency (s) of Sh and I/R groups treated with normal saline (I+Veh), different doses of gallic acid (I+GA50, I+GA100, and I+GA200) or phenytoin (I+Phen). Data are presented as mean± SEM. Symbols **p<0.01and ***p<0.001 to compare with Sh group, #p<0.05, ##p<0.01 and ###p<0.001 to compare between treated groups with I+Veh, respectively. One-way ANOVA followed by LSD post-hoc test, n=16 in each group

The initial latency (s) just 5 days after the induction of global cerebral I/R was significantly longer in all treated ischemic groups than sham operated (Sh) group (p<0.001, Figure 1). The step through latency of I/R groups after 24 h (short-term memory) significantly decreased compared with Sh group (p<0.001, Figure 2), and also was significantly higher in I+Phen than other ischemic groups treated with normal saline or GA (p<0.001). 

**Figure 3:**
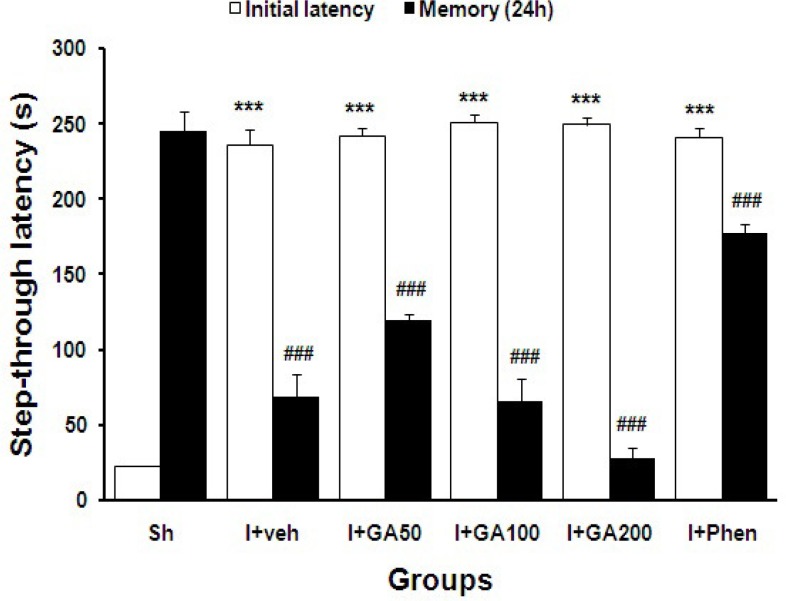
Initial and step through latencies (s) of sham operated (Sh) and I/R groups treated with normal saline (I+Veh), different doses of gallic acid (I+GA50, I+GA100, and I+GA200) or phenytoin (I+Phen). Data are presented as mean±SEM. Symbols ***p<0.001to compare initial latency with Sh group, ###p<0.001 to compare step-through latency with Sh group, respectively. Data were analyzed using paired t-test to compare initial and step-through latencies in each group, one-way ANOVA followed by LSD post-hoc test to compare initial and step-through latencies in all groups, respectively, n=16 in each group

In Figure 3 the initial and step through latencies (s) of Sh and I/R groups treated with normal saline or different doses of GA or phenytoin were compared together. Data are presented as mean±SEM (Figure 3).

**Tail flick test**

**Figure 4:**
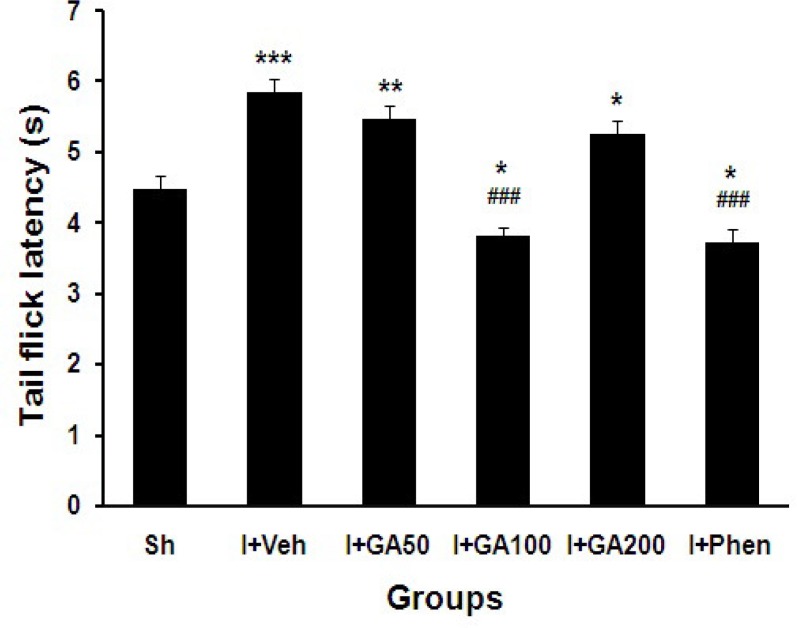
Tail flick latency (s) of sham operated (Sh) and I/R groups treated with normal saline, different doses of GA (I+GA50, I+GA100, and I+GA200) or phenytoin (I+Phen). Data are presented as mean± SEM. Symbols ***P<0.001, **P<0.01 and *P<0.05 to compare with Sh group, ###P<0.001 to compare between treated groups with I+Veh group, respectively. One-way ANOVA followed by LSD post-hoc test, n=16 in each group

Tail flick test was used to assess pain, just 5 days after induction of global cerebral I/R. Tail flick latency (s) was significantly increased in I/R groups treated with normal saline (I+Veh) and doses 50 and 200 GA compared with sham operated (Sh) group (p<0.001, p<0.01, and p<0.05, respectively). It was significantly decreased in I+GA100 and I+Phen groups compared with Sh group (p<0.001, Figure 4). 

## Discussion

According to our findings, learning and memory was impaired in I/R rats. We found that 5 days oral administration of different doses of GA before and after induction of I/R could improve short-term memory in I/R rats. Dose of 50 mg/kg of GA improved short-term memory (24 h) in passive avoidance task in I/R groups. Dose of 100 mg/kg of GA decreased pain intensity significantly in tail flick test when compared with Sh and I+Veh groups, so its effect was similarto phenytoin in I/R rats. Except in Sh group, the initial latency was increased in all ischemic groups that received GA or its vehicle. These findings show that I/R may cause the rats to avoid from going into dark chamber. 

In this work, dose of 50 mg/kg of GA improved memory deficit induced by I/R while in tail flick test dose of 100 mg/kg improved pain significantly. Animals in all groups were the same while both tests of cognition and tail flick were done as separately at different times. Our findings show that the GA may affect memory and pain with different pharmacological mechanisms. 

During the period of ischemia large quantities of stimulatory amino acids are released and calcium overload lead to an increase in free radicals that is the sign of a point called exitoxicity phase (Qian et al., 2008 ▶). Both a great production of free radicals and the deficiency or depletion of many antioxidant systems may reveal exacerbation of the oxidative cellular injury, while the supplementation of many antioxidants generates diverse outcomes (Reiter 1995 ▶; Renis et al., 1996 ▶). Many of these free radicals contain oxygen and are called reactive oxygen species (ROS). Typically, the levels of ROS and other free radicals are controlled by various scavenger molecules, known as antioxidants that are normally found within the cells which eliminate free radicals. The antioxidant defense mechanisms include antioxidant enzymes such as superoxide dismutase (SOD), glutathione peroxidase (GPx), and several non-enzymatic free radical scavengers (Murray and Stein 1989 ▶; Simon et al., 1984 ▶). The nervous tissue has a high content of polyunsaturated fatty acids (Sun et al., 2002 ▶), which are easy targets to oxidative damage by free radicals due to the unsaturated bonds they contain (Taati et al., 2012 ▶). 

On the other hand, it has been revealed that brain structures such as hippocampus which support memory are uniquely sensitive to oxidative stress due to their elevated demand for oxygen (Urso and Clarkson, 2003 ▶; Vannucci and Vannucci, 1997 ▶; Wickens 2001 ▶). Behavioral studies in animals have demonstrated that hippocampal damage can produce learning and memory impairments (Greenamyre et al., 1985 ▶; Wigstrom et al., 1986 ▶), particularly on tasks that involve place learning (Yoo et al., 2010 ▶). Therefore, it seems that hippocampus (a crucial brain area for cognition) may be damaged by transient hypoxia-reperfusion. 

It has been shown that modulation of nitric oxide (NO) availability is an important determinant of ischemic stroke risk. Thus, optimal NO/ROS balance in the brain seems to be a crucial parameter in the prevention of brain damage including ischemic stroke as well as neurodegenerative diseases (Zola-Morgan and Squire, 1986 ▶). 

Phenolic acids and flavonoids, although not essential for survival, may over the long term provide protection against a number of chronic diseases. Our recent investigation showed that oral administration of gallic acid has antioxidative activity in brain of rats with experimental neurodegenerative disorders such as Alzheimer’s and Parkinson’s diseases and decreased oxidants in main brain regions resulted in improving short- and long-term memories and motor impairment (Mansouri et al., 2012 ▶; Valizadeh et al., 2012 ▶). The main flavonoids such as anthocyanins are potent antioxidants, being able to inhibit lipid peroxidation and production of low-density lipoproteins. However, the protective effects of these compounds may not be exclusively due to their antioxidant properties and other mechanisms may also operate. Plant polyphenols have specific pharmacologic activities that interact with cell-signaling cascades, influence the cell at a transcriptional level, and down-regulate pathways that lead to cell death, rather than general properties to scavenge ROS and free radicals (Shukitt-Hale et al., 2006 ▶). 

The neuroprotective effects of many polyphenols (e.g., gallic acid) rely on their ability to permeate brain barrier and directly scavenge pathological concentration of reactive oxygen and nitrogen species and chelate transition metal ions (Tran et al., 2010 ▶). Different polyphenolic compounds were shown to have scavenging activity and the ability to activate key antioxidant enzymes in the brain and thus breaking the vicious cycle of oxidative stress and tissue damage (Koyama et al., 2010 ▶; Pande and Akoh, 2009 ▶). There is a growing interest in the potential of natural polyphenols to improve memory, learning, and general cognitive ability. Recent evidence has indicated that flavonoids may exert especially powerful actions on mammalian cognition and may reverse age-related declines (Pilsakova et al., 2010 ▶; Sarkaki et al., 2012 ▶). Since the behavioral impairments were observed after cerebral I/R, and lower dose of GA improved those deficits (Figures 1-3), it may be concluded that the GA can pass through blood brain barrier (BBB) and protect or improve neuronal function in critical brain regions involving cognition after cerebral damage by ischemia. On the other hand, ischemic/reperfusion enhances tail flick latency (inducing hypoalgesia) significantly compared with sham ischemic rats (p<0.001) and oral consumption of 100 mg/kg gallic acid could reverse it significantly (p<0.001). This effect shows gallic acid beside its antioxidative properties may affect the brain regions involving the nociception processing centrally by passing the BBB or peripherally by inhibition of the nociceptors via circulation. However, more investigations are needed to determine the mechanism of this probability. 

Gallic acid improves passive memory deficits due to I/R in male rats because of its function as antioxidant and scavenger of free radicals in injured brain tissue after I/R. However, the exact mechanisms for effect of GA on cognition and pain sense require more attentions and investigations. 
